# Factors affecting turnover intention of Nigerian employees: The moderation effect of organizational commitment

**DOI:** 10.1016/j.heliyon.2023.e23087

**Published:** 2023-12-03

**Authors:** Zanak Abet, Mohd Ashraff Mohd Anuar, Mohd Mursyid Arshad, Ismi Arif Ismail

**Affiliations:** a Department of Development and Continuing Education, Faculty of Educational Studies, Universiti Putra Malaysia, Serdang, 43400, Selangor, Malaysia

**Keywords:** Organizational commitment, Turnover intention, Theory of planned behavior, Small and medium-sized enterprises

## Abstract

Examining turnover as a noteworthy concern for businesses irrespective of their scale, this research delves into the factors influencing the inclination of employees in small and medium-sized enterprises to depart from their current workplaces. Additionally, the study explores how organizational commitment moderates the connections between attitude, subjective norms, perceived behavioral control, and intentions to leave. Methodology: Six hypotheses were formulated regarding the links between the components of the initial Theory of Planned Behavior and organizational commitment. Results: The outcomes from the partial least squares structural equation modeling indicated that the three primary predictors of the Theory of Planned Behavior have a substantial impact on turnover intention, with perceived behavioral control exerting the strongest influence. Additionally, the findings highlighted that the relationship between the Theory of Planned Behavior constructs and turnover intention is moderated by organizational commitment. Practical Implications and Originality: In this research, an expanded rendition of the Theory of Planned Behavior was employed to bring novel insights into the realm of organizational commitment among workers in small and medium-sized enterprises.

## 1 Introduction

**Fig. 1:**
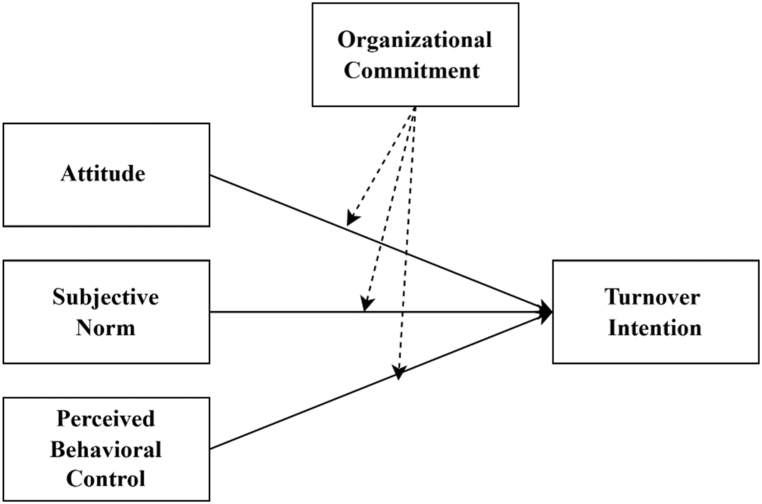
A modified version of the Theory of Planned Behavior.

Employee turnover is a critical concern for organizations in terms of its financial consequences and the disruption it causes to productivity and efficiency [1]. According to a Work Institute [2], the cost of losing an employee typically amounts to around 33 % of their base salary. According to Collins et al. [3], hiring a replacement can cost between 50 % and 75 % of an employee's pay. Such figures highlight the necessity of understanding and addressing turnover within organizations (see Fig. 1).

The negative effects of employee turnover extend beyond financial implications. When employees leave, valuable resources are diverted from the organization, decreasing efficiency and effectiveness [4]. Additionally, the costs of recruiting, training, and retaining new employees rise significantly. These consequences have been extensively studied and documented [5].

A concept that has gained considerable attention in turnover research is turnover intention. Turnover intention refers to an employee's inclination or thoughts of leaving their current organization within a specific timeframe, even if they have not taken concrete steps to leave. Several studies have examined various factors that can effectively predict turnover intention [6,7]. The Theory of Planned Behavior is an extensively utilized theoretical framework to examine behavioral intents, particularly turnover intention [8]. It is a well-established psychological theory that has been used for several decades to understand and predict human behavior. However, its age does not necessarily diminish its relevance or utility. There are several reasons why the Theory of Planned Behavior remains a valuable framework for understanding and influencing human behavior, despite its age. While the Theory of Planned Behavior may be considered “old” in terms of its origin, its enduring utility and empirical support make it a valuable tool for understanding and predicting human behavior. It continues to inform research and practical efforts aimed at promoting positive behavior change across various domains, from health promotion to environmental conservation.

According to the Theory of Planned Behavior, three main predictors—attitude, subjective norm, and perceived behavioral control—influence a person's behavioral intentions [9].

Attitude represents an individual's evaluation of favorable or unfavorable perspectives when engaging in a specific behavior [10]. It is shaped by behavioral beliefs and outcome evaluations whereby individuals assess and predict their actions' desired consequences. The second predictor, subjective norm, pertains to the influence of social norms on an individual's behavior [11]. It is influenced by how others perceive and express trust in a person's feelings and actions. Lastly, perceived behavioral control shows a person's belief in their capacity to control resources and choices while engaging in an activity.

Understanding the predictors of behavioral intention, as elucidated by the Theory of Planned Behavior model, holds significant implications for organizations aiming to address turnover issues effectively. By comprehending the factors that are linked to an employee's intention to leave, organizations can develop targeted strategies to mitigate turnover and retain valuable talent.

The Theory of Planned Behavior has been widely applied in studying various human behaviors, including employee behavior and purchasing intentions [12,13]. This study delves into the three primary predictors of the Theory of Planned Behavior and their influence on turnover intention, drawing upon existing research and exploring the potential implications for organizations seeking to improve employee retention and reduce turnover rates.

High turnover rates among small and medium-sized enterprise (SME) employees are a persistent issue in Nigeria, adversely affecting the productivity and sustainability of these businesses [14]. Previous studies consistently found a high turnover intention among SME employees nationwide [14]. This turnover trend has been identified as a contributing factor to the poor performance of SMEs in Nigeria [15]. Employee turnover has been cited as a significant reason why only a small percentage of SMEs remain operational after five years in Nigeria [14].

SMEs play a vital role in Nigeria's economy, comprising approximately 97 % of all businesses and contributing 50 % to the country's national gross domestic product [16]. However, their potential to drive economic growth and transformation is hindered by the high turnover rates among employees and the inability of managers to retain efficient personnel [17]. While employee skills and qualifications are essential, commitment to the organization is equally crucial. Committed employees are likelier to perform their jobs diligently and exceed organizational expectations [18]. Therefore, addressing the issue of employee turnover is critical for enhancing SMEs' effectiveness and overall economic growth in Nigeria. Even though a lot of research has been done on the factors that affect turnover intention, there isn't much written about the role of organizational commitment as a moderator within the framework of the Theory of Planned Behavior [9].

The turnover intention has been extensively researched in the fields of organizational psychology and human resources [19]. The existing literature on employee turnover intention has predominantly focused on individual factors such as job satisfaction [20] and job embeddedness [21]. Furthermore, the Theory of Planned Behavior has been widely adopted to comprehend the cognitive determinants of an individual's intention to engage in a particular behavior [22]. However, the use of the Theory of Planned Behavior to analyze turnover intention in Nigerian SMEs has been limited, and the impact of organizational commitment as a moderator remains unexplored.

Additionally, organizational commitment has been identified as an important factor in employee retention and turnover. Researchers have investigated the link between organizational commitment and employees' intentions to stay or leave their organizations [23]. While these factors have been extensively researched, there has been little specific examination of these factors in the context of Nigerian employees.

This study seeks to bridge this gap in the literature by examining the moderating role of organizational commitment in the relationship between key determinants of turnover intention—namely, attitude, subjective norm, and perceived behavioral control—within the context of Nigerian SMEs. While prior research has acknowledged the importance of organizational commitment in reducing turnover intention [24], few studies have integrated it into the Theory of Planned Behavior framework. Therefore, this research aims to contribute by shedding light on how organizational commitment influences the cognitive processes outlined in the Theory of Planned Behavior and, subsequently, turnover intention among SME employees in Nigeria.

The existing literature presents inconsistent findings regarding the relative strength of the three predictors of behavioral intention within the Theory of Planned Behavior framework. Some studies suggest that attitude is the strongest predictor [25]. Meanwhile, others highlight subjective norms as the most influential factor [26]. Perceived behavioral control is often reported as having the least or no significant influence on turnover intention [27]. Due to these inconsistencies, there is a need to investigate further the relationship between the three Theory of Planned Behavior predictors and turnover intention.

In light of these considerations, this study aims to explore the predictive influence of attitude, subjective norm, and perceived behavior control on turnover intention among SME employees in Nigeria. Moreover, it examines the moderating role of organizational commitment in the relationship between this Theory of Planned Behavior constructs and turnover intention. By investigating the role of organizational commitment as a potential moderator, the study seeks to illuminate the influence of individual commitment levels on turnover intention. Thus, the study's objectives are twofold: to test the association of attitude, subjective norm, and perceived behavioral control and turnover intention among SME employees in Nigeria and to investigate whether organizational commitment moderates the relationship between this Theory of Planned Behavior constructs and turnover intention. By better understanding these dynamics, organizations can develop targeted strategies to mitigate turnover and enhance employee retention in Nigeria's SME sector.

## 2 Literature review

### 2.1 Theory of Planned Behavior

This research is based on the planned behavior theory [9]. The theory offers a theoretical justification for examining turnover intention as a stand-in for real turnover and identifies behavioral intention as the main antecedent to actual action. According to the idea of planned behavior, an employee's intention is shaped by their attitude toward their workplace, the subjective norm that shapes how they perceive social pressure connected to turnover, and their view of their own power over whether to stay or quit.

### 2.2 Attitude and turnover intention

Various models have been proposed recently to explore turnover intention and its relationship to actual behavior [28]. The Theory of Planned Behavior stands out as one of the most beneficial theories, attracting attention across diverse fields and contexts. Ajzen [9] added perceived behavioral control to the Theory of Reason Action. The growth of the Theory of Planned Behavior was based on Ajzen's observation that most individual behaviors are not under complete self-imposed control. The intention to engage in a specific behavior is the immediate antecedent of that behavior following the Theory of Planned Behavior [29]. Therefore, the more individuals demonstrate the intention to perform an intended behavior, the higher the possibility they would do so [30]. The three predictors of intention in the Theory of Planned Behavior model are categorized as “attitude ", “subjective norm”, and “perceived behavioral control”. Employee attitudes are crucial in an organization because they influence their intention and performance [31]. Employees' attitudes are shaped by their favorable or unfavorable assessments of their likelihood of engaging in a given action.

Attitude is defined as an employee's positive and negative feelings toward their employer [32]. It is theorized that attitude directly affects behavioral intention. Numerous studies have demonstrated a noteworthy correlation between attitude and intention in various contexts [33]. For example, several studies on employee turnover intention confirmed that attitudes positively influence turnover intention [34]. However, other studies found that attitude negatively influences turnover intention [35]. Lei et al. [36] pointed out that employees' positive attitude toward the organization increases employees' desire to stay. From the discussion, it is anticipated that an employee's positive attitude to their current workplace will enhance their desire to remain and, consequently, reduce their turnover intention. Thus, we hypothesized that:

H1 Attitude will be negatively associated with employee turnover intention.

### 2.3 Subjective norm and turnover intention

According to Taylor and Todd [37], employees' subjective norms and compliance attitudes are influenced by their peers, significant others, and role models. Subjective norms describe how important people—coworkers, managers, family, and friends—are considered to exert social pressure or influence over one's decision to remain in or quit a job. It prompts an employee's reflections on whether the majority of those they deem significant approve of, encourage, and consent to them remaining employed [38].

Ajzen [9] stated that social and peer influences impact behavior more than personal attitudes. For instance, individuals significant to an employee can influence their decision to remain in their organization. At this stage, the employee might be compelled to comply with the significant other's expectations. Lam et al. [39] found that subjective norms negatively influence turnover intention. A recent study on the behaviors of health workers revealed that subjective norms substantially negatively influenced the organizational commitment behavior of employees [27]. Based on previous findings, this study postulated that:

H2 Subjective norms will be negatively linked to employee turnover intention.

### 2.4 Perceived behavioral control and turnover intention

Numerous researchers have examined the predictive validity of perceived behavioral control on intention and behavior [36]. Perceived behavioral control describes how simple or complex the turnover intention is thought to be to carry out. The ability of employees to perceive their own degree of control over obstacles, together with their own level of competence and self-assurance, is what ultimately keeps them with the company and lowers the likelihood of turnover. It evaluates how much people believe they can successfully manage or get beyond barriers to leaving their jobs [40].

Van Breukelen et al. [41] examined the relationship between the Theory of Planned Behavior constructs and turnover intention in the Dutch Navy; their study findings revealed no association between perceived behavioral control and turnover intention. However, previous findings have been inconsistent on the relationship between perceived behavioral control and behavioral intentions. Several studies found a robust link between perceived behavioral control and intention. For instance, Torlak et al. [27] revealed a significant association between perceived behavioral control and intention. Other studies found no relationship between perceived behavioral control and intention [42]. However, study by Shao et al. [43] confirmed a negative association with a significant negative influence of perceived behavioral control on turnover intention. Thus we hypothesized that:

H3 There is a negative association between perceived behavioral control and turnover intention.

### 2.5 Moderating role of organizational commitment

Organizational commitment has drawn most organizational scholars' attention as it appears to predict organizational outcomes like performance, organizational citizenship behavior, turnover intention, and turnover [27]. Employee loyalty, affiliation, and participation with their organization is known as organizational commitment. Since organizational commitment linked to turnover intention, it is an important variable in our study. Research has indicated that turnover intention is negatively impacted by organizational commitment [23].

To be committed to an organization means sharing its beliefs and priorities, being willing to put in significant work on its behalf, and wanting to remain a member of that organization [44]. Organizational commitment is important because it supports employees' contributions and loyalty to their organization, thereby promoting organizational development. According to Porter et al. [45], organizational commitment may be able to predict turnover. However, dedication to an organization has long been a deciding factor in whether a person chooses to stay or quit [23]. From the previous literature, organizational commitment is one of the strongest and imperative predictors in determining whether an employee will leave their current organization [46]. Furthermore, previous studies on organizational commitment revealed a strong association between organizational commitment and employee turnover intention [47]. Therefore, based on past literature, it can be concluded that organizational commitment predicts behavioral outcomes such as employee turnover intention.

Ajzen's [22] meta-analysis showed that other factors often moderate the relationship between the Theory of Planned Behavior's constructs and turnover intention. Organizational commitment serves as a moderator in various contexts. For instance, in a meta-analysis on the antecedents of employee turnover, Griffeth et al. [48] revealed that commitment moderates employee job attitude and turnover intention relationship. Moreover, the importance of organizational commitment in understanding employee behavior and attitudes is widely acknowledged [49]. Furthermore, according to Mobley et al. [50], commitment might influence turnover intention and, consequently, turnover behavior. Additionally, recent research showed that commitment has a major association with employee behavior and attitudes [51]. Therefore, organizational commitment can be utilized within the Theory of Planned Behavior model as a moderator to improve the correlation between the Theory of Planned Behavior constructs and turnover intention. Additionally, the Theory of Planned Behavior is open to modification and expansion by introducing a moderator or antecedent variables [9]. Despite substantial research on the direct association between the three basic predictors and intentions [34], findings are inconsistent regarding the relationship between these constructs and turnover intention [41]. Although organizational commitment may directly linked to work-related outcomes, it may be more beneficial to consider it as a construct that facilitates the effects of other factors on turnover intention. Therefore, organizational commitment can be used as a moderator in the Theory of Planned Behavior to improve the predictive influence of the Theory of Planned Behavior constructs and turnover intention. Thus, this study hypothesized that:

H4 Organizational commitment moderates the association between attitude and employee turnover intention.

H5 Organizational commitment moderates the association between subjective norms and employee turnover intention.

H6 Organizational commitment moderates the association between perceived behavioral control and employee turnover intention.

## 3 Methodology

### 3.1 Participants and procedures

Self-administered questionnaires were employed to gather quantitative data, with the study population comprising employees of manufacturing Small and Medium-sized Enterprises (SMEs) in Lagos state, Nigeria. A stratified random sampling technique was used to select the study's respondents to enhance the representativeness of the various population segments [52]. After conducting the stratified random sampling, subjects from each stratum were chosen randomly to take part in the study. The questionnaires contained an introductory letter explaining the study's purpose. The data was finally collected from the respondents within three months and 3weeks (March 2022–July 2022) with the aid of two enumerators. A grand total of 420 questionnaires were disseminated, and 366 were subsequently returned. However, after thorough checks, only 330 questionnaires were considered useable for analysis. Thus, the response rate in this study was 78.6 %.

### 3.2 Measurements

The questionnaire contained questions about demographic variables, the Theory of Planned Behavior's constructs, organizational commitment, and turnover intention. The survey instrument consisted of 45 questions designed to test the study's variables. Each item was derived from prior research and adjusted to align with the specific objectives of the current research investigation. There were five latent variables in this study. The items were measured on a seven-point Likert scale ranging from 1 (strongly disagree) to 7 (strongly agree). Six items from Bothma and Roodt [53] study were used to ascertain turnover intention. Organizational commitment was measured by nine items from Mowday et al.'s [44] Organizational Commitment Questionnaire. For the Theory of Planned Behavior's constructs, employees' attitude was measured using six items; subjective norm had five items; and five questions were utilized to measure perceived behavioral control, all adapted from Kim and Han's study [54]. The questionnaires were completed anonymously to ensure confidentiality.

### 3.3 Ethical consideration

The research received ethical approval from the Universiti Putra Malaysia Ethics Committee for Research Involving Human Subjects (JKEUPM), under reference number JKEUPM-2021-413. Informed consent was sought before participant recruitment by the researcher. An exception to the requirement for parental consent was made since the study posed no danger to the participants. The employees were made aware that participation was completely optional and that they had the option of agreeing or declining.

### 3.4 Data analysis

The respondents' demographics were described using descriptive statistics. The application of PLS-SEM was chosen to assess model fit and test the hypotheses presented, as advocated by Hair et al. [55] for its suitability in testing models across a spectrum of complexities and accommodating small to medium sample sizes. Given its capacity to assess the predictive association between attitude, subjective norms, perceived behavioral control, and turnover intention, PLS-SEM was employed for this study. It also makes it possible to examine how organizational commitment modifies the association between mindset, subjective standard, perceived behavioral control, and intention to leave.

### 3.5 Missing value

The data underwent scrutiny and screening through basic frequency distribution and descriptive statistics. Any out-of-range or incorrectly coded values were promptly identified. A frequency test was run on each variable to identify incorrect and missing values. The SPSS analysis showed that there were no missing values for the 330 questionnaires. As a result, the data entry is given correctly without missing and incorrect values. Therefore, the 330 valid questionnaires retrieved were deemed fit for data analyses.

### 3.6 Common method bias

To evaluate the existence of common method bias, we conducted an assessment using the heterotrait-monotrait (HTMT) ratio and inner variance inflation factor values. In line with the guidance provided by Nitzl et al. [56], a correlation exceeding r > 0.90 signifies the potential presence of common method bias among the primary constructs. However, the investigation yielded correlation values among the constructs, all below 0.90, as indicated in the HTMT Table 5 below. Notably, the highest observed correlation value was 0.581, indicating the absence of common method bias in our study. Following the thresholds recommended by Kock [57] scrutinized the inner variance inflation factor values, where a variance inflation factor exceeding 3.30 indicates potential model contamination due to common method bias. This study's highest variance inflation factor value observed in the structural model assessment table was merely 1.561. This value falls substantially below the established threshold of 3.30, confirming that our study lacks common method bias.

## 4 Results

### 4.1 Profile of respondents

The demographic variables were measured considering five items: gender, age groups, educational level, length of work, and monthly income. According to the results on the demographics of the respondents, approximately 63.64 % were male (n = 210), and approximately 36.36 % were female employees (n = 120). Respondents were also categorized into five age groups according to their age range: younger than 20, 20 to 30, 31 to 40, 41 to 50, and above 50. In this survey, participants were requested to choose an age range. The result indicates that the respondents' age 4.55 % (n = 15) are from the range less than 20 years of age, which is the lowest. The age 41.21 % (n = 136) is from 21 to 30 years, which is high in representation; age 32.73 % (n = 108) is from 31 to 40 years, the age 17.58 % (n = 58) is from 41 to 50 years, and age 3.94 % (n = 13) is from above 50 years in representation. The demographic profile was not included in further analysis in this study (Table 1).

**Table 1:** Demography profile.

Demographic Profile	Categories	Frequency	Percentage (%)
Gender	Male	210	63.64
	Female	120	36.36
Age	Less than 20	15	4.55
	20–30	136	41.21
	31–40	108	32.73
	41–50	58	17.58
	Above 50	13	3.94
Educational Level	SSCE	56	16.97
	Diploma	100	30.30
	Bachelor	118	35.76
	Masters	53	16.06
	PhD	3	0.91
Years in organization	Less than 1 year	44	13.33
	1–3 years	115	34.85
	4–6 years	67	20.30
	7–9 years	38	11.52
	10 years above	66	20.00
Monthly income (₦)	Less than 20,000	47	14.24
	21,000–30,000	44	13.33
	31,000–40,000	19	5.76
	41,000–50,000	58	17.58
	Above 50,000	162	49.09

### 4.2 Descriptive analysis

Table 2 depicts the five latent variables' mean scores and standard deviation. The range of mean scores for the variables, which extends from 4.67 to 5.35, reveals that all variables were evaluated favorably (out of a seven-point scale).

**Table 2:** Descriptive results of constructs.

Construct	Mean	SD
TOI	5.04	1.06
OC	5.01	1.01
ATT	4.67	1.32
SN	4.94	1.02
PBC	5.35	1.11

**Notes**: **TOI** = Turnover intention; **OC** = Organizational commitment; **ATT** = attitude; **SN** = subjective norm; **PBC** = perceived behavioral control.

### 4.3 Measurement model

The measurement model was tested using both Exploratory Factor Analysis (EFA) and Partial Least Squares Structural Equation Modeling (PLS-SEM) Confirmatory Factor Analysis (CFA). EFA was employed to examine the factor structure of all 54 components. In the initial stages of EFA, principal components analysis (PCA) with varimax rotation was utilized. The Kaiser-Meyer-Olkin (KMO) score of 0.79 and the findings of Bartlett's test of sphericity (χ^2^ = 1755.94; p = 0.000) suggested that the data were suitable for EFA. The Kaiser criteria were utilized to ascertain the number of components, considering only those with eigenvalues exceeding items with loadings greater than 0.60. Consequently, three entries with factor loadings below 0.6 were excluded from the analysis. The findings of the study indicated that attitude, with a value of (0.037), subjective norm (0.052), and organizational commitment (0.031), exert a minor impact on turnover intention. In contrast, perceived behavioral control, with a value of (0.167), has a moderate effect on turnover intention, surpassing the 0.15 % threshold.

As per the PLS-SEM output, the CFA model demonstrated a satisfactory fit with the data (SRMR = 0.067; NFI = 0.908 [55]). When analyzing the latent variable associations, significant connections were identified among the components (p < 0.01). Convergent validity was confirmed as all extracted Average Variance (AVE) values exceeded 0.5, and all factor loadings surpassed 0.70 (see Table 3). Moreover, internal consistency was evident with Composite Reliability (CR) ratings ranging from 0.873 to 0.953. Lastly, all constructs exhibited Rho_A values exceeding 0.7 [58].

**Table 3:** Construct reliability and validity.

Constructs	rho_A	AVE	CR	α
ATT	0.884	0.827	0.966	0.958
OC	0.895	0.798	0.973	0.968
PBC	0.824	0.831	0.961	0.949
SN	0.922	0.791	0.950	0.934
TOI	0.977	0.844	0.970	0.963

Notes: TOI = Turnover intention; OC = Organizational commitment; ATT = attitude; SN = subjective norm; PBC = perceived behavioral control; AVE = average variance extracted; CR = composite reliability.

This investigation utilized the variance inflation factors to explore multicollinearity. A variance inflation factor of more than 5 indicates multicollinearity. The results of this investigation indicated that the most significant inner variance inflation factors value is 1.561, and the lowest variance inflation factors value is 1.004, showing no multicollinearity in the data (Table 4) [59].

**Table 4:** Multicollinearity test for exogenous latent constructs.

Construct	1	2	3	4
1. ATT	1.561			
2. OC	1.004			
3. PBC				1.286
4. SN			1.026	
5. TOI				

Note. TOI = Turnover intention; OC = Organizational commitment; ATT = attitude; SN = subjective norm; PBC = perceived behavioral control.

**Table 5:** Measurement model: discriminant validity- Fornell–Larcker criterion.

Constructs	1	2	3	4	5
1. ATT	0.909				
2. OC	0.561	0.893			
3. PBC	0.006	−0.015	0.912		
4. SN	−0.087	−0.125	0.136	0.889	
5. TOI	−0.194	−0.141	−0.212	−0.125	0.919

Note. TOI = Turnover intention; OC = Organizational commitment; ATT = attitude; SN = subjective norm; PBC = perceived behavioral control. The diagonal represents the square root of AVE, whereas the off-diagonal numbers represent correlations between latent variables.

In PLS-SEM, establishing discriminant validity is a critical step in ensuring that latent constructs in research are distinct. Discriminant validity assesses the extent to which one concept differs from another using empirical criteria. This study used the Fornell-Larcker criteria, suggested by academics, as a method to integrate different methodologies [59]. The fulfillment of the Fornell-Larcker criteria was confirmed by observing that the square root of the AVE for each construct exceeded the correlation coefficients between any pair of constructs. This verification indicated the attainment of discriminant validity, as supported by the findings presented in Table 5.

According to Henseler et al. [58], a typical recommendation is to strive for HTMT levels below the cutoff of 0.90. In the PLS-SEM context, discriminant validity has been sufficiently established when HTMT values are below this cutoff. Values above 0.90 may be an indication of measurement or model specification problems as well as a potential lack of discriminant validity. Table 6 displays that the results are below the threshold of 0.09, which indicates that discriminant validity was achieved.

**Table 6:** Results of heterotrait-monotrait ratio (HTMT).

Constructs	ATT	OC	PBC	SN	TOI
ATT					
OC	0.581				
PBC	0.054	0.044			
SN	0.110	0.138	0.152		
TOI	0.202	0.138	0.217	0.132	

Note. TOI = Turnover intention; OC = Organizational commitment; ATT = attitude; SN = subjective norm; PBC = perceived behavioral control. The diagonal represents the square root of AVE, whereas the off-diagonal numbers represent correlations between latent variables.

### 4.4 Structural model

The R^2^, Q^2^, and significance of paths were employed to assess the structural model. The R^2^ result of the Theory of Planned Behavior model revealed that the total explained variance of turnover intention is 9 %, indicating a weak predictive accuracy. However, the variance explained in turnover intention when the moderating variable (organizational commitment) was included in the model reached 15 %. This is 6 % higher than the original model. This finding indicates a satisfactory influence of organizational commitment in moderating the factors predicting the model. In this study's path model, the predictive relevance Q^2^ of turnover intention has a value of 0.120, suggesting that the model has predictive relevance. Since the Q^2^ value is greater than zero, the model is well-fitted and has high predictive relevance.

The direct effect of the Theory of Planned Behavior's three basic predictors on turnover intention was confirmed as hypothesized. As displayed in Table 7, attitude is significantly associated with turnover intention (β = −0.146, t = 2.349, p = 0.019). Hence, H_1_ was supported. In addition, the subjective norm is substantially linked to turnover intention (β = −0.140, t = 2.422, p = 0.016). Therefore, H_2_ was supported. Finally, perceived behavioral control robustly negatively influenced turnover intention (β = −0.202, t = 3.558, p = 0.000). Therefore, H_3_ was supported.

**Table 7:** Result for assessing the structural model.

Path	OS/Beta	SM	SD	Confidence Interval 95 % Bias Corrected		T	P
				LL	UL		
ATT → TOI	−0.146	−0.143	0.062	−0.285	−0.040	2.349	0.019
SN → TOI	−0.140	−0.144	0.058	−0.247	−0.023	2.422	0.016
PBC → TOI	−0.202	−0.200	0.057	−0.321	−0.099	3.558	0.000

Note: TOI = Turnover intention; OC = Organizational commitment; ATT = attitude; SN = subjective norm; PBC = perceived behavioral control. OS= Original sample SM= Sample mean SD= Standard deviation.

### 4.5 The moderating test of organizational commitment

It was hypothesized in this study that organizational commitment would moderate the relationship between the Theory of Planned Behavior constructs and turnover intention. A moderator is a third factor that modifies the relationship between the independent and dependent variables [60]. The moderating effect in structural models can be analyzed through several means. Bootstrapping was used in this study to observe the moderating effect. The study's findings show that organizational commitment significantly moderates the Theory of Planned Behavior constructs and turnover intention. The results in Table 8 reveal each Theory of Planned Behavior's constructs't and p values. The results showed attitude and turnover intention with a t-value of (2.430) and a p-value of (<0.015), followed by subjective norm and turnover intention with a t-value of (2.033) and a p-value of (<0.043). Lastly, perceived behavioral control and turnover intention had a t-value of (2.012) and a p-value of (<0.045).

**Table 8:** Indirect path.

Hypotheses	Beta	T	P
ATT × OC → TOI	−0.139	2.430	0.015
SN × OC → TOI	−0.107	2.033	0.043
PBC × OC → TOI	−0.120	2.012	0.045

Note. TOI = Turnover intention; OC = Organizational commitment; ATT = attitude; SN = subjective norm; PBC = perceived behavioral control.

## 5 Discussions

This study tested the hypothesis that the three fundamental predictors of the Theory of Planned Behavior significantly have association with turnover intention among SME employees and that organizational commitment moderates the relationship between the Theory of Planned Behavior's constructs and turnover intention. The results revealed that the SME employee turnover intention mainly depends on their attitude toward the organization, subjective norm, and perceived behavioral control, among which perceived behavioral control and subjective norm had the highest association with turnover intention. When SME employees have positive attitudes towards their organization and perceive more favorable factors in the organization, their turnover intention will decrease. This indicates that employees' positive attitude towards their organization and their sense of behavioral control and efficacy over situations or circumstances in the organization are more relevant for forming behavioral intentions to remain in the organization.

Remarkably, this study's findings revealed perceived behavioral control as the best predictor of turnover intention among the three basic predictors of the Theory of Planned Behavior. This discovery contrasts Ajzen's Theory of Planned Behavior, which asserted that attitude is the strongest component in predicting intention and that perceived behavioral control has a less predictive effect on intention [9]. Similarly, some studies on turnover intention found a lower or insignificant relation between perceived behavioral control and turnover intention [61]. However, perceived behavioral control emerged as the strongest predictor of turnover intention. The result demonstrates a significant negative association with turnover intention. This finding aligns with Hilverda et al.'s [62] study, which found perceived behavioral control to be the strongest predictor of employee voice among the three basic predictors of the Theory of Planned Behavior. This finding may be attributable to the study's varied cultural environment, which also influenced the employees' perceived behavioral control and their intention to leave their jobs [63].

This study's findings on the three predictors of the Theory of Planned Behavior align with previous studies, which found that one's attitude, subjective norm, and perception of control significantly influence turnover intention [64]. In a study on teachers' intentions to leave their jobs, Costan et al. [34] found that perceived behavioral control was the most critical factor in determining teaching intentions out of the three basic predictors based on the Theory of Planned Behavior. Furthermore, social influences, especially from those with direct power over employees (e.g., spouses, parents, mentors, supervisors, colleagues, and family members), substantially linked to the likelihood of an employee's decision to leave their current workplace. Finally, the employee's ability to control situations at work could lead to a high level of perceived behavioral control and a low turnover intention. This suggests that SME employees must gain the required control reflected by a supportive organization, which will subsequently reduce their turnover intention.

This study revealed that organizational commitment robustly negatively moderates the relationship between the three basic predictors of the Theory of Planned Behavior and turnover intention. The findings indicated that attitude had the most significant association with turnover intention when organizational commitment moderates the relationship between the Theory of Planned Behavior predictors and turnover intention. The study's findings revealed that attitude with a high level of organizational commitment negatively linked to employees' turnover intention (B = −0.139, p < 0.015). Considering the results, it may be affirmed that a positive attitude will lead to lower turnover intention when there is a high level of organizational commitment. **S**ubjective norm was found to influence turnover intention (B = −0.107, t = 2.033). This implies individual people are likelier to intend to engage in an activity if they sense social pressure from most of their referents to act accordingly. Likewise, in perceived behavioral control, the intention to leave the organization would decrease with any improvement in the employees' view of behavioral control. Employees who are committed to the organization and feel they have control over work situations could have high perceived behavioral control, ultimately reducing turnover intention.

Theory of Planned Behavior's three basic predictors negatively associated with turnover intentions, and organizational commitment significantly moderates the relationship between attitude and turnover intention, subjective norm and turnover intention, and perceived behavioral control and turnover intention. It was concluded that those employees who have favorable attitudes and subjective norms and also perceive a high level of control would be more committed to their organization, thus reducing turnover intention. The endogenous variable turnover intention's R^2^ was 0.150 when the moderator was included in the model, indicating that the exogenous variables explained 15.0 % of the variation, which is moderate [65].

## 6 Conclusion

This study proposed and evaluated an empirical model investigating SME employees' turnover intention in Nigeria. To explain SME employees' turnover intention, Ajzen's [9] Theory of Planned Behavior model was expanded to ascertain the variations of the turnover intention with organizational commitment as a moderator between the Theory of Planned Behavior's three basic predictors and turnover intention. This study's contributions are as follows. Firstly, the addition of organizational commitment as a moderator to the Theory of Planned Behavior is one of the study's key theoretical contributions. While the Theory of Planned Behavior is a well-established paradigm for analyzing behavioral intentions, it primarily focuses on determinants at the individual level. The integration of organizational commitment enhances the theory. This supplement gives a more in-depth look at the processes underpinning turnover intention in the context of Nigerian SMEs. This research also underlines the study's practical relevance. It gives actionable insights into the SME sector by explaining the moderating impact of organizational commitment within the Theory of Planned Behavior framework. This not only advances theory but also directly assists practitioners by providing suggestions on how to improve organizational commitment as a means of minimizing employee turnover intention. Secondly, the three fundamental predictors of the Theory of Planned Behavior predict the SME employee's turnover intention, with the perceived behavioral control construct having the greatest influential predictive effect (f^2^ = 0.167). Thus, policymakers must focus on initiatives that enhance SME employees' perception of control by providing a supportive work environment to assist employees in controlling circumstances in the organization. Management should encourage a family-like atmosphere by facilitating and developing good relationships and strong bonds between management and employees and among colleagues through social activities such as recreation. Also, management should foster a healthy work environment to develop good employee attitudes by communicating the organization's goals, mission, values, and culture to help employees adapt to the work environment. Lastly, another notable finding is that commitment moderates the relationship between the Theory of Planned Behavior's predictors and turnover intention. Employees' commitment to the organization reduces turnover intention. Therefore, management should formulate policies and implement effective practices that bind employees and make them feel emotionally obligated and committed to the organization. By extending the Theory of Planned Behavior with a novel moderator, contextualizing it within the Nigerian SME environment, reaffirming its contemporary relevance, and proposing practical solutions for firms, this work contributes to the field of organizational behavior. Collectively, these theoretical contributions improve our knowledge of turnover intention in SMEs and increase the theoretical basis of behavioral research. In concluding, by evaluating the Theory of Planned Behavior model in a frontline staff sample from Nigerian manufacturing SMEs, this study contributes to the understanding of employee turnover intention. The findings offer specific and practical suggestions that factory managers may implement to improve organizational performance by decreasing expenses related to employee attrition and increasing workforce stability. Thinking about turnover from a psychological angle broadens the scope of management theory and application. All things considered, this study offers significant theoretical and practical insights that are beneficial to management scholars and industry executives.

## 7 Limitations and direction for future studies

In addition to the highlighted contributions, the current study also includes several significant limitations. The study employed a self-administered survey, an approach that does not provide in-depth comprehension of the topic studied. A more in-depth understanding of the issue at hand would be possible by utilizing a mixed-method approach, which entails employing both qualitative and quantitative research approaches to investigate the factors that influence the intention of the employee to leave an organization. This study did not attempt to measure actual employee turnover. Instead, it focused on employees' intentions. It is recommended that subsequent studies track the behavioral prediction power of the employed Theory of Planned Behavior model. Future research should also consider using longitudinal designs to examine how extensively the Theory of Planned Behavior predicts SME employee turnover intention.

## 8 Implications

Legislators ought to concentrate on programs that augment the sense of control held by SME workers by furnishing a nurturing workplace that aids staff in managing situations within the company. By supporting and cultivating positive relationships and strong links between management and staff as well as among coworkers through social activities like recreation, management may promote a family-like environment. To help workers adjust to their new work environment, managers should also promote a positive work environment by sharing the organization's objectives, mission, values, and culture. Through engagement, recognition, and cultural activities, management may enhance employee attitudes and lower attrition. The association between the Theory of Planned Behavior's predictors and turnover intention is also moderated by commitment, which is another noteworthy discovery. The loyalty of employees to the company lowers turnover intention. As a result, management needs to create procedures and regulations that tie workers and give them a sense of emotional commitment to the organization.

## CRediT authorship contribution statement

**Zanak Abet:** Visualization, Software, Conceptualization. **Mohd Ashraff Mohd Anuar:** Writing – original draft. **Mohd Mursyid Arshad:** Supervision. **Ismi Arif Ismail:** Resources.

## Declaration of generative AI and AI-assisted technologies in the writing process

In the process of preparing this work, the author(s) utilized ChatGPT to enhance the academic language. Following the use of this tool/service, the author(s) thoroughly reviewed and edited the content as necessary and assume(s) full responsibility for the publication's content.

Note: Upon reasonable request, the corresponding author is willing to furnish the data supporting the results of this study.

## Declaration of competing interest

The authors declare that they have no known competing financial interests or personal relationships that could have appeared to influence the work reported in this paper.
