# Supplementary material for: Factors affecting turnover intention of Nigerian employees: The moderation effect of organizational commitment
Source: Heliyon. 2023 Dec 3;10(1):e23087. doi: 10.1016/j.heliyon.2023.e23087 (PMC10754884; doi:10.1016/j.heliyon.2023.e23087)
Supplement: Multimedia component 1 [file mmc1.pdf]

# Questionnaire On The Moderating Effect Of Organizational Commitment On The Relationship Between Individual Factors And Employee Turnover Intention Of Small And Medium Enterprises In Nigeria.


**By**

**Zanak Samuel Abet**

**GS53811**

Dear sir/ Madam,

I am Zanak Samuel Abet, a doctoral candidate specializing in Human Resource Development at the Faculty of Educational Studies, University Putra Malaysia. I am currently researching the Moderating effect of organizational commitment on the relationship between individual factors and employee turnover intention of Small and Medium Enterprises in Nigeria.

You have been selected to participate in the study because you are an employee of a Small and medium enterprise (SME) in Nigeria. Therefore, you are in a position to provide the appropriate information required for the study. All you have to do is set aside 10 minutes to complete this questionnaire. Please do not participate in the survey if you are not an employee of a registered manufacturing SME. Your contribution will help expand the existing body of knowledge on employee turnover intention among Nigerian SMEs. Please, note that the information you provide will be used solely for academic purposes and kept confidential. Hence there are no risks involved in participating in the study.

Thank you,

Researcher: Zanak Samuel Abet

zeenak.zanak01@gmail.com

+60163184908

**<u>Section A: Demographic</u>**

Would you please check the appropriate box to answer the following demographic questions that best describe you?

1. Gender

☐Male ☐Female

2. Age (years)…………………………………….

3. What is the highest level of your education?

☐Below secondary school ☐SSCE ☐Diploma ☐Bachelor ☐Masters ☐PhD

4. For how long have you been working in this organization………………………..

5. Monthly income ₦………………………………………………………..

**Section B**

**Part 1: Questions on turnover intention**

In regards to how you feel about your current organization, please indicate the degree of your agreement or disagreement with each statement by ticking each item with an appropriate scale.

| **Sr. #** | **Construct items** | **Strongly Disagree** | **Disagree** | **Slightly Disagree** | **Undecided** | **Slightly Agree** | **Agree** | **Strongly Agree** |
|---|---|---|---|---|---|---|---|---|
| 6 | I often considered leaving my job. | | | | | | | |
| 7 | My current job satisfies my personal needs. | | | | | | | |
| 8 | I will probably look for a new job soon | | | | | | | |
| 9 | I often do dream about getting another job that will better suit my personal needs. | | | | | | | |
| 10 | I will leave this organization as soon as possible. | | | | | | | |
| 11 | I often look forward to another day at work | | | | | | | |

**Section B**

**Part 2: Questions on organizational commitment**

Would you please read the following statements and indicate your level of agreement with each statement by ticking the appropriate box? Responses range from 1 strongly disagree to 5 strongly agree.

| **Sr. #** | **Construct items** | **Strongly Disagree** | **Disagree** | **Slightly Disagree** | **Undecided** | **Slightly Agree** | **Agree** | **Strongly Agree** |
|---|---|---|---|---|---|---|---|---|
| 12 | I am willing to put in a great deal of effort to help this organization be successful | | | | | | | |
| 13 | This organization is a great organization to work for | | | | | | | |
| 14 | This organization deserves my loyalty | | | | | | | |
| 15 | My ideas and that of the organization are similar. | | | | | | | |

| Sr. # | Construct items | Strongly Disagree | Disagree | Slightly Disagree | Undecided | Slightly Agree | Agree | Strongly Agree |
|---|---|---|---|---|---|---|---|---|
| 12 | I am willing to put in a great deal of effort to help this organization be successful | | | | | | | |
| 16 | I am proud to tell others that I am part of this organization. | | | | | | | |
| 17 | This organization inspires me to bring out my best. | | | | | | | |
| 18 | I am happy I chose this organization to work for over others I was considering when I joined. | | | | | | | |
| 19 | I often do not agree with this organization's policies on important issues that affect its employees. | | | | | | | |
| 20 | I care about this organization's future. | | | | | | | |

**Section B**

**Part 3: Questions on Attitude**

Would you please indicate the level to which you agree with the following statement?

| Sr. # | Construct items | Strongly Disagree | Disagree | Slightly Disagree | Undecided | Slightly Agree | Agree | Strongly Agree |
|---|---|---|---|---|---|---|---|---|
| 21 | My intention to leave this organization is a bad idea | | | | | | | |
| 22 | It will be unpleasant for me to leave this organization | | | | | | | |
| 23 | For me, to leave this organization is unwise | | | | | | | |
| 24 | For me, to leave this organization will be extremely favorable | | | | | | | |
| 25 | My intention to leave this organization is undesirable | | | | | | | |
| 26 | My intention to leave this organization will be extremely good for me. | | | | | | | |

**Section B**

**Part 4: Questions on subjective Norm**

Would you please indicate the degree to which you agree with the following statement?

| Sr. # | Construct items | Strongly Disagree | Disagree | Slightly Disagree | Undecided | Slightly Agree | Agree | Strongly Agree |
|---|---|---|---|---|---|---|---|---|
| 27 | Most people who are important to me think I should leave this organization. | | | | | | | |
| 28 | Most people who are important to me would want me to leave this organization. | | | | | | | |
| 29 | People whose opinions I value would prefer that I leave this organization | | | | | | | |
| 30 | Most people who are important to me think it would be a good idea for me to leave this organization | | | | | | | |
| 31 | Most people who are important to me will approve of my leaving this organization. | | | | | | | |

**Part 5: Perceived Behavioural control**

Would you please indicate the level to which you agree with the following statement?

| Sr. # | Construct items | Strongly Disagree | Disagree | Slightly Disagree | Undecided | Slightly Agree | Agree | Strongly Agree |
|---|---|---|---|---|---|---|---|---|
| 32 | Whether or not I leave this organization is entirely up to me. | | | | | | | |
| 33 | I am confident that if I want, I can leave this organization | | | | | | | |
| 34 | For me, leaving this organization would be very difficult | | | | | | | |
| 35 | I have control over my decision to leave this organization | | | | | | | |
| 36 | I have better opportunities and resources if I leave this organization. | | | | | | | |

**Part 6: Organizational trust**

Would you please indicate the level to which you agree with the following statement?

| Sr. # | Construct items | Strongly Disagree | Disagree | Slightly Disagree | Undecided | Slightly Agree | Agree | Strongly Agree |
|---|---|---|---|---|---|---|---|---|
| 37 | The organization's management is honest in its efforts to understand the employees' perspectives. | | | | | | | |
| 38 | This organization has a poor future unless it can employ competent management | | | | | | | |
| 39 | I trust the management of this organization to make good decisions for the future of the organization. | | | | | | | |
| 40 | This organization will be willing to deceive the employees to gain an advantage. | | | | | | | |
| 41 | I am confident that this organization will always try to treat me fairly | | | | | | | |
| 42 | The management of this organization seems to be effective. | | | | | | | |

**Part 7: Perceived organizational support scale**

Would you please indicate the level to which you agree with the following statement by ticking the statement that suits your perception?

| Sr. # | Construct items | Strongly Disagree | Disagree | Slightly Disagree | Undecided | Slightly Agree | Agree | Strongly Agree |
|---|---|---|---|---|---|---|---|---|
| 43 | My organization cares about my well-being. | | | | | | | |
| 44 | My organization strongly considers my goals and values. | | | | | | | |
| 45 | My organization shows little concern for me | | | | | | | |
| 46 | My organization cares about my opinions | | | | | | | |
| 47 | My organization is willing to help me if I need a special favor | | | | | | | |

| Sr. # | Construct items | Strongly Disagree | Disagree | Slightly Disagree | Undecided | Slightly Agree | Agree | Strongly Agree |
|---|---|---|---|---|---|---|---|---|
| 43 | My organization cares about my well-being. | | | | | | | |
| 48 | If given a chance, my organization would take advantage of me | | | | | | | |
| 49 | My organization would forgive an honest mistake on my part. | | | | | | | |
| 50 | I can get help from my organization when I have a problem | | | | | | | |
